# The effect of attribute framing on beliefs and attitudes toward branded and generic medications

**DOI:** 10.1007/s10865-025-00562-1

**Published:** 2025-03-13

**Authors:** Emily K. Spotts, Kelly S. Clemens, Kate Faasse, Andrew L. Geers

**Affiliations:** 1 Department of Psychology, University of Toledo, 2801 West Bancroft, Toledo, OH 43606 USA; 2 Department of Psychology, Illinois State University, Normal, IL USA; 3 School of Psychology, University of New South Wales, Sydney, NSW Australia

**Keywords:** Framing, Generic, Brand status, Attitudes, Treatment beliefs, Communication

## Abstract

Generic medications are developed to match brand-name medications in terms of active ingredients, quality, safety, and strength. Because generic medications cost less than their brand-name counterparts, they present an opportunity to reduce financial burden for patients and social institutions. Studies show, however, that patients often possess negative beliefs and evaluations of generic medications, thereby undermining use and effectiveness. In two pre-registered online experiments with community adults (Total *N* = 750), we tested the effect of attribute framing on attitudes, attitude certainty, and effectiveness beliefs regarding generic and brand-name medications. In both experiments, framing (positive vs. negative) and brand status (generic vs. brand name) of an allergy medication were manipulated in a 2 × 2 between-participant design. The results of both experiments produced strong effects of the framing and brand status manipulations. Specifically, allergy medicines were rated more favorably with a positive frame as compared to a negative frame. Also, branded allergy medicines were rated more positively than generic allergy medicines. In contrast with the pre-registered hypotheses, the two manipulated variables did not reliably interact to predict outcomes. These results held constant across multiple sets of stimuli and dependent measures. The findings support the position that positive framing can be employed to improve positive evaluations and effectiveness beliefs for both over-the-counter generic and branded medications.

**Supplementary Information:**

The online version contains supplementary material available at 10.1007/s10865-025-00562-1.

## Introduction

Rising healthcare costs are an ever-growing challenge in modern medicine. In the United States, this problem was the impetus for healthcare reforms such as the 2010 Affordable Care Act. Despite such changes, the challenge of escalating healthcare costs remains. In 2021, the United States spent $4.3 trillion on healthcare services, averaging approximately $12,900 per person, an increase of 2.7 percent (Trends in Health Care Spending, 2023). Recent reports suggest that nearly 4 in 10 Americans struggle to afford their prescription medications, with nearly 40% discontinuing or altering their treatment regimen to save money (Guttentag, 2023).

One strategy to manage some of the rising healthcare costs is the use of cheaper generic medicines as an alternative to branded options. Generic medications are pharmaceutical products that have the same active ingredient as the equivalent branded medication (Alrasheedy et al., 2014). Generics, however, are estimated to cost between 20 and 90% less than their branded alternatives (Dunne et al., 2013). Generics are also required to be the same strength, pharmaceutical form, and route of admission as the branded counterparts. Although there can be differences in terms of inactive ingredients, color, shape, and most often, price, the medications undergo rigorous registration and strict requirements to ensure the treatment is of a good quality and safety rating, improving the dependability of the medicine. Generic medicines must be considered pharmaceutically bioequivalent to their branded counterparts, meaning they have clinical equivalence, thus preventing the preclinical and clinical testing that is required of the original brand (Galgatte et al., 2014). This saves the producer money and allows for the decreased prices on generic medications that are reflected to buyers (Howland, 2009).

Although generic medications have the same therapeutic effects as branded medications and should therefore be interchangeable in the eyes of both prescribers and patients, a large body of research has suggested that this is not always the case. Patients do not always find generics to be an equal alternative treatment, with medicine consumers holding misconceptions and negative attitudes about generic medicines across the globe (Al Ameri et al., 2011; Babar et al., 2010). For example, a review of 52 studies found that 35.6% of laypeople viewed generics as less effective than branded medication (Colgan et al., 2015). These negative perceptions are found across countries and health care systems (Dunne et al., 2013; Gebresillassie et al., 2018) Negative attitudes and misconceptions about effectiveness are major obstacles that prevent patients from choosing and employing generic medicines to treat their ailments (Faasse et al., 2013; Himmel et al., 2005). Even being aware that generic medications are biologically equivalent does not necessarily result in participants forming a positive opinion of the medication, with patients reporting that branded medications were still more effective or that generic medicines caused more side effects (Keenum et al., 2012).

One strategy for increasing the acceptance of generic medicines is by altering how these drugs are presented. In particular, it may be possible to alter perceptions and use of generic medications by employing attribute framing. In attribute framing, the emphasis on a single attribute in a message is altered to present the message in a more positive or negative manner (Tversky & Kahneman, 1981). Often, the information is presented in terms of gains (a positive frame) or presented in terms of losses (a negative frame). Importantly, other than this emphasis difference, the message remains equivalent. For example, in a study by Wilson et al. (1987), significantly more participants were willing to engage in surgery when the risks were presented with a positive frame, a 10% probability of surviving, as compared to when the risks were presented with a negative frame, a 90% probability of dying. Since the initial studies on attribute framing, similar results have been found across a variety of domains, including product evaluations (Levin & Gaeth, 1988), feeling states (Stark et al., 2017), condom use (Linville et al., 1993), cancer screening (Ferrer et al., 2012), and influenza vaccines (O’Connor et al., 1996). The possible beneficial effects of attribute framing have particular importance in healthcare and medical treatments, as treatments have long-term effects on a person’s life and well-being (Barnes et al., 2019; Gallagher & Updegraff, 2012; Mao et al., 2021).

There is reason to believe that positive framing could be particularly advantageous for improving perceptions of generic medications. This is because research has found that framing effects are often stronger with unfamiliar stimuli as compared to familiar stimuli (Levy et al., 2020; Smith & Wortzel, 1997). This may be because those who are familiar with an object people rely on their prior knowledge to form future judgments and evaluations and are less likely to engage with and integrate information inconsistent with this prior knowledge. Unfamiliar objects do not have this prior knowledge network, so these are more likely to benefit from framed information (Han et al., 2009). For example, a recent study found that the impact of framing on COVID-19 booster vaccine intentions was moderated by vaccine familiarity. Specifically, positive framing increased vaccination intentions over negative framing when the vaccine under consideration was an unfamiliar vaccine, as compared to a familiar vaccine (Barnes & Colagiuri, 2022). Although familiarity does not always moderate framing effects (Barnes et al., 2023; Chen & Chang, 2016), research suggests that, because generic medications receive less exposure and enjoy little name recognition as compared to branded medications, they are less familiar, and thereby may benefit more from positive framing (Han et al., 2009). While generic medicines are not inherently negatively framed, the combination of the general adverse opinions of generic medicines with a lack of positive advertising otherwise presented about branded medicines about treatment efficacy could be detrimental to the presentation of use and acceptance of generic medicines. Further, health information comes from a variety of sources, including Internet social media sites, which have become an increasingly popular way in which people are receiving information about health treatments (Afful-Dadzie et al., 2023; Lin & Kishore, 2021). Social media sites can provide an array of perspectives on different medicines, so it is likely that people are exposed to differing opinions and types of messaging about treatments. Thus, it is possible that the impact of this negative bias on attitudes and beliefs of effectiveness can be reduced by employing positive framing.

## Present research

Two studies examined the influence of attribute framing on attitudes and effectiveness beliefs towards generic and branded medicines. Both studies were conducted using allergy medicines. Allergies are common, with 25.7% of American adults being diagnosed with a seasonal allergy condition (Ng & Boersma, 2023). Allergy medicines are frequently used to relieve symptoms, with 75% of allergy sufferers using over-the-counter allergy medicine (Consumer Healthcare Products Association, 2017). Further, there are many branded allergy medicines with generic alternatives on the market, and an increasing number of people are choosing over-the-counter allergy medicines compared to prescription medicines.

Based on the prior framing literature, we expected a main effect of attribute framing, such that positive framing would increase effectiveness beliefs and favorability of evaluations as compared to negative framing. We also anticipated a main effect of brandedness, such that effectiveness beliefs and evaluations would be higher for the branded medication as compared to the generic medication. Finally, we anticipated an interaction, such that the effect of attribute framing would be most pronounced for generic medications.

In addition to assessing attitudes and beliefs regarding medication effectiveness, we also measured attitude certainty. Certainty refers to a subjective assessment of conviction individuals hold about their attitudes (Rucker et al., 2014). Certainty is a meta-cognitive assessment (e.g., “is my evaluation of the medication valid?”) that occurs after an initial evaluation (e.g., “what is my evaluation of the medication?”). Recent theory suggests that persuasive communications can sometimes alter certainty, even if they do not alter attitudes (Rucker et al., 2014; Tormala, 2016). Further, research finds that attitudes held with greater certainty are more likely to alter subsequent behavior (e.g., Bechler et al., 2021; Conner & Norman, 2022) and that measures of attitude certainty are influenced by framing manipulations (Rucker et al., 2008). As such, in the present research we measured attitude certainty along with beliefs of effectiveness and attitudes to assess if any observed effect of attribute framing on branded and generic medications occurs also on this meta-cognitive judgment.

## Study 1

### Methods

#### Participants and design

Based on a review of attribute framing studies conducted by Barnes et al. (2019), an average effect size (*f* = 0.17), with alpha set at 0.05, and power of 90%, a minimum number of 366 participants was estimated using G*Power (Faul et al., 2007). In this study, 375 community participants were recruited through Prolific, an online recruitment platform. Participants were required to be over the age of 18, fluent in English, and reside in the United States at the time of their participation. All participants provided informed consent through an electronic form. Participants ranged in age from 18 to 78 (*M*_*age*_ = 34.78, *SD* = 13.39). The sample was 49.1% female, 49.1% male, and 1.9% identifying as another gender or preferring not to disclose their gender. Participants lived in the United States at the time of the survey completion. Participants were 74.9% White, 8.8% Hispanic, 7.2% Asian, 5.6% African American, 2.9% Multi-racial, and 0.5% Middle Eastern. Participants were compensated for participating in the study. The study was pre-registered on the Open Science Framework (https://osf.io/se36g) and approved by the University of Toledo Institutional Review Board.

Participants were randomly assigned to one of the four conditions using Qualtrics simple randomization tool. The design was a 2 (attribute frame type: positive vs. negative) by 2 (medication type: generic vs. branded) between-subjects factorial design.

#### Materials and measures

Study stimulus materials and measures were displayed using the Qualtrics survey platform. Following consent, participants were presented with a single medication stimulus screen. Participants were randomly assigned to view one of four stimulus medications. The stimuli included a picture of the medication along with a text description (see text descriptions by condition in Table 1). Two of the medications were well-known branded medicines: Claritin and Zyrtec, which are common allergy medicines in the United States and are both considered second-generation antihistamines. In an effort to control for extraneous factors (e.g., the physical appearance of the packaging), we created fictitious generic medications (e.g., “Home and Health”), and designed packages that mirrored the appearance of the branded equivalents. The text accompanying the images of the medications indicated that they were brand name or generic medicines, respectively. Two of the medications were fictitious allergy medicines that were designed to mirror the physical appearance of the branded equivalents, but the text indicated they were generic medications. We included two sets of branded and generic medication stimuli so that the results would not be particular to one specific brand of medication. Preliminary analyses indicated that the results were similar across the stimuli and thus we collapsed across the two variations in data analysis.

**Table 1:** Framing condition by medication type for Studies 1 and 2

Medication type	Frame	Paragraph
*Study one*		
Branded	Positive	The United States Food and Drug Administration has recently announced that in clinical trials, the name-brand medication Claritin RediTabs have been found to reduce allergy effects for approximately 74% of individuals. That is, 74 out of 100 people experience reduced allergy symptoms after taking **Claritin RediTabs**
Generic	Positive	The United States Food and Drug Administration has recently announced that in clinical trials, the generic brand Home and Health Allergy Relief Tablets have been found to reduce allergy effects for approximately 74% of individuals. That is, 74 out of 100 people experience reduced allergy symptoms after taking **Home and Health Allergy Relief Tablets**
Branded	Negative	The United States Food and Drug Administration has recently announced that in clinical trials, the name-brand medication Claritin RediTabs have been found to show no reduction in allergy effects for approximately 26% of individuals. That is, 26 out of 100 people experience no reduction in allergy symptoms after taking **Claritin RediTabs**
Generic	Negative	The United States Food and Drug Administration has recently announced that in clinical trials, the generic brand Home and Health Allergy Relief Tablets have been found to show no reduction in allergy effects for approximately 26% of individuals. That is, 26 out of 100 people experience no reduction in allergy symptoms after taking **Home and Health Allergy Relief Tablets**
*Study two*		
Branded	Positive	The United States Food and Drug Administration has recently announced that in clinical trials, the name-brand medication **Benadryl Allergy Liqui-Gels** has been found to reduce allergy effects for approximately 74% of individuals. That is, 74 out of 100 people experience reduced allergy symptoms after taking **Benadryl Allergy Liqui-Gels**
Generic	Positive	The United States Food and Drug Administration has recently announced that in clinical trials, the generic medication **Cabinet: Allergy Relief** has been found to reduce allergy effects for approximately 74% of individuals. That is, 74 out of 100 people experience reduced allergy symptoms after taking **Cabinet: Allergy Relief**
Branded	Negative	The United States Food and Drug Administration has recently announced that in clinical trials, the name-brand medication **Benadryl Allergy Liqui-Gels** has been found to show no reduction in allergy effects for approximately 26% of individuals. That is, 26 out of 100 people experience no reduction in allergy symptoms after taking **Benadryl Allergy Liqui-Gels**
Generic	Negative	The United States Food and Drug Administration has recently announced that in clinical trials, the generic medication **Cabinet: Allergy Relief** has been found to show no reduction in allergy effects for approximately 26% of individuals. That is, 26 out of 100 people experience no reduction in allergy symptoms after taking **Cabinet: Allergy Relief**

Underlined text indicates the information that varied across conditions

The bold text indicates the generic or branded medication names

Orthogonal to the branded/generic manipulation was the attribute framing manipulation. That is, participants were randomly assigned to have the description of the medication presented with either a positively or negatively framed statement about the efficacy of the medication (see Table 1 for the information presented in each condition). Following the manipulations, participants completed measures of medication attitudes, medication effectiveness beliefs, attitude certainty, and manipulation checks, followed by demographic items.

*Medication attitudes*. Participant attitudes towards the treatment were assessed using 10 items on 10-point semantic differential scales with attributes at the endpoints. The items, taken from past advertisement research (Briñol et al., 2004; Tormala & Petty, 2002), were: good/bad, unpleasant/pleasant, negative/positive, useful/useless, beneficial/harmful, ineffective/effective, harmful/beneficial, worthless/valuable, low quality/high quality, and unhelpful/helpful. These items were averaged together and showed good internal validity, α = 0.96.

*Medication effectiveness beliefs.* An examination of the treatment perception literature revealed a variety of scales assessing patient treatment expectations, drug attitudes, and health beliefs (De Las Cuevas & de Leon, 2019; Devilly & Borkovec, 2000; Sowunmi, 2022). Existing measures, however, are broad, and do not directly assess effectiveness beliefs or include items specific to unknown treatments, which was not the focus of the present set of experiments. As such, we developed a set of items to specifically capture effectiveness beliefs for allergy medications. The items were designed to focus on the effectiveness of allergy medications to reduce allergy symptoms and to include the name of a medication, so as to constrain responses specifically to the particular medication presented in the stimulus materials. Here, perceptions of treatment effectiveness were measured using seven items, including "I believe that [medication name] tablets are an effective medication for allergy symptoms", “I expect [medication name] to work in the future”, and “The experience of allergy symptoms would be completely resolved after using [medication name]”. All items are provided in supplemental materials. These items were scored on a Likert-type scale ranging from 1 *(strongly disagree*) to 10 (*strongly agree*) and averaged. The resulting measure displayed strong internal validity, α = 0.97.

*Attitude certainty.* To assess attitude certainty, we used three items derived from past research (Barden & Petty, 2008). A sample item is "How certain are you of your feelings toward [medication]?" These items were scored on a Likert-type scale ranging from 1 to 10 and the items were averaged to create a feeling of certainty scale, which demonstrated good internal validity, α = 0.85. It should be noted that in pre-registration, we planned to have six certainty items, but ultimately only three certainty items were obtained and included. This change is acknowledged in a protocol deviation report posted in the Open Science Framework.

*Manipulation checks.* We included two items to assess if the manipulations were effective. First, we included a single item to assess the success of the framing manipulation. At the end of the study we asked participants to identify the percentage of individuals who had reduced allergy symptoms when taking the medication they read about. Participants were able to select between four numerical options (e.g., 66%) and the option of “I do not recall.” Based on whether participants correctly selected the percentage that was presented to them, we created a dichotomous score of “correct” or “incorrect”, that served as the framing manipulation check. The second manipulation check item was used to determine if the branded medications were indeed perceived as more familiar than the generic medications. In this, we asked participants to rate their level of familiarity with the medication they read about earlier in the study. This was scored on a Likert-type scale ranging from 1 (*extremely unfamiliar*) to 7 *(extremely familiar*).

*Demographics.* Participants answered demographic items, including gender, age, ethnic background, and income.

*Overview of analysis.* Table 2 provides the means and standard deviations of all measures by condition. The pre-registered analysis plan for the continuous dependent measures was to enter them into separate 2 (Frame Type) × 2 (Medication Type) between-subject analysis of variance (ANOVA). Preliminary analyses revealed many analyses violated the assumption of homogeneity of variance (i.e., Levene’s test was significant). As such, we also tested each dependent variable using the Welch one-way ANOVA and Games-Howell effect tests, which do not assume equal variances. As expected for ANOVA, which is robust to this violation, the analyses yielded few differences between the standard and the Welch ANOVAs and Games-Howell tests. For ease of presentation and consistency with the pre-registered analysis plan, here we present the results of the standard ANOVAs and directly note the instances when the Welch ANOVA and Games-Howell tests produced different results. Further, medication attitudes and effectiveness beliefs were unexpectedly highly correlated (*r*. = 87), suggesting the scales likely correspond to a single latent construct. Here, for completeness, we present the pre-registered separate analyses and posthoc analyses from combining the two scales (a deviation report is provided in the Open Science Framework for the change in analytic plan). Finally, controlling for demographic variables (age, gender, ethnicity, and income) did not alter the results of either Study 1 or 2, and thus covariates are not included in analyses.

**Table 2:** Means and standard deviations (in parentheses) for dependent variables in Study 1 by condition

	Generic			Branded		
Measures	Positively framed (*N* = 94)	Negatively framed (*N* = 95)		Positively framed (*N* = 92)	Negatively framed (*N* = 94)	
Medication attitudes	8.01 (1.08)	5.82 (1.82)		8.26 (0.99)	6.38 (1.83)	
Medication effectiveness	6.98 (1.19)	4.31 (2.16)		7.34 (1.50)	5.18 (2.35)	
Attitude certainty	7.55 (1.60)	7.31 (1.84)		7.88 (1.49)	6.86 (1.94)	
Frame manipulation check	0.88 (0.32)	0.96 (0.20)		0.97 (0.18)	0.90 (0.30)	
Brand manipulation check	1.91 (1.40)	1.51 (1.17)		5.72 (1.30)	5.51 (1.54)	

Medication attitudes, medication effectiveness, and attitude certainty are on 10-point scales, with higher values reflecting more positive attitudes, effectiveness ratings, and certainty, respectively. Frame manipulation check is a dichotomous variable, with 0 = incorrect response and 1 = correct response. Brand manipulation check is on a 7-point scale, with higher scores indicating greater familiarity with the brand

### Results

#### Manipulation checks

In Study 1, 93% of participants correctly identified the frame information at the end of the study and this was not statistically significant by condition, χ^2^ (3) = 7.05, *p* = 0.07. Next, when familiarity scores were subjected to a 2 (Frame Type) × 2 (Medication Type) between-subject ANOVA, there was a strong Medication Type main effect, *F*(1, 371) 773.52, *p* < 0.001, partial η^2^ = 0.68. The branded medication was rated as more familiar (*M* = 5.61) than the generic medication (*M* = 1.71). The ANOVA also produced a weaker effect of Frame Type, *F*(1, 371) 4.82, *p* = 0.03, partial η^2^ = 0.01, but no significant two-way interaction, *F*(1, 371) 0.52, *p* = 0.47, partial η^2^ < 0.01. The medication was perceived as somewhat more familiar with the positive frame versus the negative frame. The Medication Type effect was also observed in the Welch ANOVA. Notably, the Frame Type effect did not remain when tested with the Welch ANOVA, *F*(1, 372.84) 1.47, *p* = 0.23. Taken together, these data support the perspective that participants recognize the message frame and found the branded medication more familiar than the generic medication.

#### Medication attitudes

To examine the influence of the two manipulations on medication attitudes, scores on the attitude scale were subjected to a 2 (Frame Type) × 2 (Medication Type) between-subjects ANOVA. As hypothesized, this analysis yielded a significant effect of Frame Type, *F*(1, 371) 174.85, *p* < 0.001, partial η^2^ = 0.32. Positive frame participants held more favorable attitudes about the allergy medication (*M* = 8.13) than did negative frame participants (*M* = 6.10). The main effect of Medication Type was also significant, *F*(1, 371) = 6.99, *p* = 0.01, partial η^2^ = 0.02, indicating that attitudes were more positive toward the branded medication (*M* = 7.31) as compared to the generic medication (*M* = 6.91). Contrary to hypotheses, the two-way interaction was not significant, *F*(1, 371) 0.979, *p* = 0.32, partial η^2^ < 0.01. The same outcomes were found with Welch and Games-Howell tests.

#### Medication effectiveness beliefs

When medication effectiveness belief scores were submitted to a 2 (Frame Type) × 2 (Medication Type) ANOVA, we observed a significant Frame Type main effect, *F*(1, 371) 157.52, *p* < 0.001, partial η^2^ = 0.30. Participants given the positive frame believed the medication would be more effective (*M* = 7.16) than those given the negative frame (*M* = 4.74). The Medication Type main effect was also significant, *F*(1, 371) 10.28, *p* = 0.001, partial η^2^ = 0.03. This effect shows that the branded medication was expected to be more effective (*M* = 6.25) than the generic medication (*M* = 4.31). Similar to the attitude scores, the two-way interaction was not significant, *F*(1, 371) = 1.74, *p* = 0.19, partial η^2^ < 0.01. The same outcomes were found with Welch ANOVA and Games-Howell tests.

#### Medication effectiveness perceptions

As attitude and belief scores were highly correlated (*r* = 0.87), we conducted an unplanned analysis with a scale created by averaging items of both scales (α = 0.98). The scores were subjected to a 2 (Frame Type) × 2 (Medication Type) between-subjects ANOVA, that yielded a significant effect of Frame Type, *F*(1, 371) = 187.66, *p* < 0.001, partial η^2^ = 0.34, with positive frame participants having more favorable perceptions about the allergy medication (*M* = 7.73) than negative frame participants (*M* = 5.51). The main effect of Medication Type was again significant, *F*(1, 371) = 8.19, *p* < 0.005, partial η^2^ = 0.02, with perceptions being more positive for the branded medication (*M* = 6.84) than the generic medication (*M* = 6.39). The interaction term remained non significant, *F*(1, 371) = 1.03, *p* = 0.31, partial η^2^ < 0.01.

#### Attitude certainty

Similar to the belief and attitude scores, a 2 (Frame Type) × 2 (Medication Type) ANOVA on the certainty scores produced a significant main effect of the Frame Type, *F*(1, 371) = 12.54, *p* < 0.001, partial η^2^ = 0.03. Those in the positive frame condition held their attitudes toward the allergy medication with greater certainty (*M* = 7.72) than individuals given the negative frame (*M* = 7.09). The main effect of Medication Type was not statistically significant, *F*(1, 371) 0.11, *p* = 0.74, partial η^2^ < 0.001. The Frame Type by Medication Type interaction was significant, *F*(1, 371) 0.4.88, *p* = 0.03 partial η^2^ < 0.01. Effect tests found that when given the branded medication, participants were more certain in their attitude with the positive frame (*M* = 7.88) as compared to the negative frame (*M* = 6.86), *t*(371) = 4.06, *p* < 0.001, *d* = 0.42. Unexpectedly, the positive frame (*M* = 7.55) and the negative frame (*M* = 7.31) groups did not differ for the generic medication, *t*(371) = 0.17, *p* = 0.35, *d* = 0.02. The Welch ANOVA and Games-Howell tests yielded similar results for the certainty scores.

### Study 1 Discussion

A pre-registered experiment tested whether the effects of attribute framing on medication attitudes, beliefs, and attitude certainty are influenced by branded/generic status. Confirmatory analyses revealed a strong influence of framing on the three dependent measures. Analyses also revealed an effect of medication type: the branded medication was perceived more favorably and effective as compared to the generic medication. These analyses, however, found no evidence for the hypothesis that message frames have a stronger influence on generic medications as compared to branded medications. Moreover, analyses of the attitude certainty measure that found a frame-by-medication type interaction indicated the nature of this interaction was not as anticipated. Specifically, although attitude certainty scores were numerically higher for the positive frame across medication types, the positive frame was only significantly larger than the negative frame for the branded medication. It is not clear why this unexpected result occurred. It is possible that this unanticipated effect was spurious and is not replicable. Study 2 provided an opportunity to test this effect a second time.

Another unexpected finding was the strong correlation between the medication attitude and effectiveness belief items. A review of the scales suggests that the attitude items (e.g., harmful/beneficial) likely captured evaluations of medication effectiveness. Moreover, as participants were presented a limited set of information to derive their assessments, it seems reasonable that there was little cause for divergence in attitude and effectiveness beliefs. In Study 2 we combined the attitude and effectiveness belief items to form a single medication effectiveness perception dependent measure.

## Study 2

Study 2 provides a second test of whether branded status influences the effect of framing on medication perceptions. The design was similar to that of Study 1 with two exceptions. First, in Study 1, the stimuli included two generic and two branded medications which did not differ significantly in terms of participant perceptions. To increase generalizability, in Study 2 we included a new set of generic and branded medicine stimuli. Second, and more notably, the generic medication stimulus used in Study 2 was not designed to look identical to the branded medication, as it was in Study 1. That is, in Study 1 the generic medication stimuli were fake and designed to look nearly identical to the branded medication. This was done so we could manipulate generic vs. branded status separately from other factors, such as packaging appeal. This, however, could have influenced the results of Study 1. Specifically, as participants were familiar with the branded medication, they were likely also familiar with the packaging. As a consequence, participants may have felt positive feelings or exhibited an implicit positive bias toward the generic medication because the packaging felt familiar. Thus, it could be argued that the familiar packaging weakened the influence of the framing manipulation on the generic medication and thereby hindered the anticipated frame-by-medication type interaction. Further, generic medicines often have different packaging than branded equivalents, so visually similar generic and branded medicines may limit ecological validity (Greene, 2014). To address these issues, in Study 2 we used a generic medication with more standard generic packaging features; ones that did not mirror the branded medication. Finally, as the results of the attitude certainty measure were unexpected, Study 2 allows for a second test of this finding.

### Methods

#### Participants design

We relied on the same power analysis calculation used in Study 1. As such we recruited 375 community participants through Prolific. Participants were required to be over the age of 18, fluent in English, and reside in the United States at the time of their participation. Participants ranged in age from 18 to 78 (*M*_*age*_ = 35.47, *SD* = 13.31). The sample was 56.8% female, 40.5% male, and 2.7% identifying as another gender or preferring not to disclose their gender. Participants lived in the United States at the time of the survey completion. Participants were 65.1% White, 18.4% Asian, 10.1% Hispanic, 9.3% African American, 1.9% Multi-racial, 1.3% Native American, and 0.5% Middle Eastern. Participants were compensated for participating in the study. The experiment was pre-registered on the Open Science Framework (https://osf.io/pr9mk) and approved by the University of Toledo Institutional Review Board.

Participants were randomly assigned to a 2 (attribute frame type: positive vs. negative) by 2 (medication type: generic vs. branded) between-subjects factorial design.

#### Materials and measures

Study 2 was designed to replicate Study 1 with new medication stimuli. The branded medicine photo and information were replaced with Benadryl, another well-recognized over-the-counter allergy medicine. The generic medicine photo and information were replaced with the Amazon generic brand “Cabinet”. This medicine packaging includes black-and-white plain text of “Allergy Relief” with pink and white striped borders. Further, the effectiveness and attitude measures were highly correlated (*r* = 0.87) in Study 1, suggesting that the two measures are likely not capturing different constructs. Thus, these two measures were combined in Study 2 to form a 17-item “Medication Perceptions” scale, which demonstrated strong internal validity (α = 0.97). All materials and measures in the survey otherwise remained the same.

### Results

Means and standard deviations by condition of the attitude, belief, and attitude certainty dependent measures are presented in Table 3. In Study 2, the standard ANOVA tests and the Welch ANOVA and Games-Howell tests did not produce divergent results.

**Table 3:** Means and standard deviations (in parentheses) for dependent variables in Study 2 by condition

	Generic			Branded		
Measures	Positively framed (*N* = 95)	Negatively framed (*N* = 92)		Positively framed (*N* = 93)	Negatively framed (*N* = 95)	
Medication perceptions	7.71 (0.97)	5.81 (1.94)		7.94 (1.31)	6.55 (1.95)	
Attitude certainty	7.52 (1.53)	6.89 (1.31)		8.20 (1.51)	7.45 (1.81)	
Frame manipulation check	0.87 (0.33)	0.90 (0.30)		0.91 (0.28)	0.96 (0.20)	
Brand manipulation check	1.96 (1.66)	1.73 (1.56)		5.96 (1.34)	5.85 (1.39)	

Medication attitudes, medication effectiveness, and attitude certainty are on 10-point scales, with higher values reflecting more positive attitudes, effectiveness ratings, and certainty, respectively. Frame manipulation check is a dichotomous variable, with 0 = incorrect response and 1 = correct response. Brand manipulation check is on a 7-point scale, with higher scores indicating greater familiarity with the brand

#### Manipulation checks

In this data set, 86% of participants identified the correct frame information at the end of the study and this did not differ by condition, *χ*^2^ (3) = 4.35, p = 0.23. Next, when familiarity scores were subjected to a 2 (Frame Type) × 2 (Medication Type) between-subject analysis of variance (ANOVA), the analysis produced the anticipated Medication Type main effect, *F*(1, 371) = 697.42, *p* < 0.001, partial η^2^ = 0.65. Participants in the branded medication condition (*M* = 5.90) reported greater familiarity than the generic medication condition (*M* = 1.84). There was no Frame Type main effect, *F*(1, 371) = 1.17, *p* = 0.28, partial η^2^ < 0.01, or two-way interaction, *F*(1, 371) = 0.17, *p* = 0.69, partial η^2^ < 0.01.

#### Medication perceptions

When medication perceptions scores (α = 0.97) were subjected to a 2 (Frame Type) × 2 (Medication Type) between-subjects ANOVA, it yielded a significant effect of Frame Type, *F*(1, 371) = 81.06, *p* < 0.001, partial η^2^ = 0.18. Positive frame participants held more favorable perceptions about the allergy medication (*M* = 7.70) than did negative frame participants (M = 6.19). The main effect of Medication Type was also significant, *F*(1, 371) = 12.95, *p* < 0.001, partial η^2^ = 0.03, with perceptions being more positive for the branded medication (*M* = 7.24) than the generic medication (*M* = 6.65). As was found in Study 1, the two-way interaction was not significant, *F*(1, 371) = 0.63, *p* = 0.43, partial η^2^ < 0.01.

#### Attitude certainty

Similar to the belief and attitude scales, a 2 (Frame Type) × 2 (Medication Type) ANOVA on the certainty scores (α = 0.77) produced a significant main effect of the Frame Type, *F*(1, 371) 18.27, *p* < 0.001, partial η^2^ = 0.05. Those in the positive frame condition held their attitudes toward the allergy medication with greater certainty (*M* = 7.86) than individuals given the negative frame (*M* = 7.18). The main effect of Medication Type was also statistically significant, *F*(1, 371) = 14.90, *p* < 0.001, partial η^2^ < 0.04, such that participants who received information about a branded medicine held their attitudes with greater certainty (*M* = 7.82) than participants who received information about a generic medicine (*M* = 7.21). The two-way interaction was non-significant, *F*(1, 371) = 0.12, *p* = 0.73 partial η^2^ < 0.001.

### Study 2 discussion

The results of Study 2 conform closely to those of Study 1. Again, attribute framing influenced responses, such that medications were rated as more positive and attitudes were held with greater certainty with a positive frame as compared to a negative frame. Also, Study 2 found branded medications were viewed more positively and attitudes more certain as compared to generic medications. Unlike Study 1, Study 2 did not find an interaction between frame and medication type on the attitude certainty measure. Further, consistent with Study 1, we found no evidence that the effect of frame is stronger for generic medications. Thus, the results generally replicate those of Study 1 with a different set of stimulus material, including a generic medication stimulus that looked different from the branded medication. The conceptual replication of the Study 1 results are valuable as they increase generalizability and also as they rule out an explanation for the of an interaction between brandedness and framing in Study 1 based on the design of the stimuli. That is, we designed the packaging of the generic medication in the stimulus material in Study 1 to appear nearly identical to that of the branded medication. This visual similarity to a well-known branded medication may have increased positive feelings about the generic medications and thereby weakened the influence of the framing manipulation. As the results of Study 2 were similar to those of Study 1, and in this case the generic medication stimuli did not share similarities with a branded medication, it indicates that this stimulus similarity explanation cannot account for the lack of a brandedness X framing interaction.

## General discussion

The goal of the present study was to assess the combined influence of attribute framing and brand on perceptions of medicines in terms of effectiveness, attitudes, and feelings of certainty. Two pre-registered experiments manipulated both attribute framing and branding status and both found main effects of framing and branding, such that allergy medicines were rated more positively after being presented with positively-framed information as compared to negatively-framed information, and branded allergy medicines were rated more positively than generic allergy medicines. These findings were generally consistent across three dependent measures and three different sets of stimuli.

Consistent with hypotheses, both of the studies revealed robust main effects of framing, such that those who received positively framed information had more positive attitudes, higher certainty, and greater beliefs of medication efficacy. This is in line with prior literature on framing effects, which has established that items described using positive framing are generally perceived as superior to items described using negative framing (Levin & Gaeth, 1988). The magnitude of the effects produced by the framing manipulation were large in size. These results support the position that positive framing should be used when medical professionals and advertisers describe either branded or generic medicines to consumers. Similarly, both studies found a main effect of brandedness, such that participants consistently rated branded medicines more positively than generic medicines. This effect occurred across different brands and different packaging presentations. This is consistent with prior literature, which has established more favorable attitudes and opinions towards branded medicines. Notably, the effects of the medication type manipulation were generally smaller in magnitude, as compared to that of the framing manipulation. Thus, we conclude that the studies were both well-powered to detect small effects and that, whereas both manipulations shifted medication perceptions, framing had the greater overall influence.

The primary goal of the present study was to determine if there is an interaction between brandedness and frame type. Prior research has found this in some cases, framing effects are stronger when evaluating objects and contexts that are unfamiliar rather than familiar (Barnes et al., 2022). Since generic medicines are typically less familiar to participants, we hypothesized that brandedness and framing type would interact, with the benefit of positive framing being larger for generic medications. The manipulation check data showed that the framing type was recognized by participants and generic medicines were rated as significantly less familiar. However, the interaction was not found in either study with any set of stimuli. These results instead suggest that framing effects do not diverge based on brandedness status. Thus, inconsistent with hypotheses, the significance tests did not show a specific advantage of using positive framing for generic medicines.

It is presently unclear why there was a lack of an interaction between framing and brandedness. One possibility is that the interaction was present, however the analyses failed to detect this effect. In the analyses, the magnitude of effect for the interaction term (partial eta square) produced values of less than 0.01, and several below 0.001. Given that the a priori power calculation estimated a sample size for power at 90%, and that the analyses were able to detect the small effects of the mediation type main effect, we believe the study appropriately failed to reject the null hypothesis that framing and medication type interact in altering medication effectiveness perceptions. Additionally, it should be noted that the factors of framing and familiarity do not always interact. For example, a recent study by Barnes et al. (2023) did not find a significant interaction between familiarity with COVID-19 vaccine brand and attribute framing.

Another possible explanation for the obtained pattern of results is that the participants were generally familiar with allergy medicines and the symptoms associated with allergy conditions, which therefore limited the unfamiliarity effects of generic medicines which the interaction relies on. Thus, it may be that to obtain a stronger framing effect due to medication unfamiliarity, multiple factors are involved, not simply familiarity with the evaluation object. It may also rely on other relevant decision factors, including other qualities of the treatment and decision factors not specific to the treatment, such as illness and symptoms. This possibility is consistent with explanations that suggest multiple attributes of a judgment are integrated and thereby drive attribute framing effects (e.g., Seta et al., 2017). From such perspectives, it is not just familiarity of the medicine that would alter feelings of uncertainty and thus strengthen the framing effect. Instead, multiple factors play a role in influencing uncertainty, such as the type of illness being treated, the resulting symptoms expected, and other treatment-related attributes. For example, ambiguity of produce quality has been found to moderate framing effects (Hoch & Ha, 1986), and it is possible that framing effects will be stronger for generic treatments when their quality is also ambiguous. Also, framing may be more influential if individuals have a low level of familiarity with allergy symptoms as well as evaluating a generic allergy medication. In sum, it may be that framing and brandedness do interact in some cases, such as when a medication is used to treat a new medical condition or one with novel symptoms. Future research should assess this and related possibilities. At the moment, our data suggest that for commonly known illnesses of low severity, framing may not differentially influence generic and branded medication perceptions.

Although the results did not show an advantage of positive framing for generic medications as compared to branded, they do show that, in general, positive attribute framing may be a useful tool to improve the perceptions of all medicines, including generic options. That is, positive framing does improve perceptions of generics, which is beneficial, as generic medicines are significantly cheaper than branded alternatives while being biologically equivalent and having identical pharmaceutical effects. This lowered cost allows the consumer to save money on necessary medicine, which is crucial as the average spending on medicine increases every year. Further, as some Americans are abandoning their medicine use as a result of cost, it is critical to decrease medical spending when possible (Mykyta & Cohen, 2023). Employing positive framing to improve perceptions of generics could potentially be used to alter patient outcomes. For example, prior research suggests that the perceptions of reduced effectiveness of generic medications are associated with lower treatment adherence (Faasse et al., 2013). As positive framing has been found to change health behavior (Gallagher & Updegraff, 2012), it may be possible to strategically use positive message framing to increase adherence to generic medications. Future research should examine this possibility.

In both studies, we measured attitude certainty along with effectiveness perceptions. Recent theory suggests that communications sometimes change meta-cognitive assessment of certainty, even if they do not change initial attitudes (Rucker et al., 2014; Tormala, 2016). In both studies, framing altered attitude certainty, such that participants reported greater certainty for positively framed messages. These results are consistent with studies on political attitudes that found message framing to alter attitude certainty (Bizer et al., 2011). Certainty has been characterized as an indicator of attitude strength, such that attitudes held with greater certainty are expected to last longer, are more likely to resist counter-persuasion, and are more likely to guide behavior than attitudes held with less certainty (Tormala & Rucker, 2018). In terms of the present context, this finding suggests that framing medications positively could not only change initial perceptions but also impact thoughts and behavior in subsequent situations due to elevated certainty. This possibility warrants future investigation. The findings with attitude certainty were less consistent with the brandedness variable. Whereas Study 1 found framing and branding to interact such that the benefit of brand status was seen in only the negative frame condition, Study 2 found the benefit of brand status across frame conditions. Given the mixed findings with branded status on attitude certainty, additional studies are required to further examine this relationship. However, taken together the results suggest a benefit of brandedness on attitude certainty. Given that attitudes are stronger when held with greater certainty, and that strong attitudes are more likely to guide behavior, it may be that this elevated level of certainty is one reason brand status impacts behavioral outcomes such as medication adherence. Future research is required to examine this possibility.

There are several opportunities for future research to build upon these results to ensure the replicability of results and true lack of an interaction between frame type and brandedness. One potential method to adapt the current materials by implementing another form of framed information, such as by alternatively framing cost information as compared to efficacy information (e.g., “[insert medication name] is 84% cheaper than the brand-name equivalent”. This has ecological validity as one of the main benefits of the use of generic medicines is cost-saving abilities. Additionally, rather than assessing participant perceptions of medicine efficacy, the present study design could be altered to instead measure expected side effects from the medicine. This is of particular benefit, as side effect expectations can be linked to actual increases in the experience of side effects, a psychobiological phenomenon known as the nocebo effect (Colloca, 2024). Finally, there are likely several psychological factors that can be used to better understand the relationship between brandedness and attribute framing, such as one’s perceived sensitivity to medicine (Horne et al., 2013) or overall perceptions of generic medicines. For example, those who have an increased perceived sensitivity to medicine may benefit more from the effects of positive message framing, as negatively framed health information could build upon these perceptions of sensitivity and cause the individual to believe they are at a greater risk for experiencing adverse side effects. Further, those with decreased overall perceptions of generic medicines could be of decreased benefit from attribute framing, as these attitudes are more strongly held and can be therefore more difficult to change through simple communication techniques. These possibilities warrant evaluation in future work.

### Strengths and limitations

The present set of pre-registered studies were well-powered and used multiple sets of stimuli, showing consistent results on measures of attitudes and, perceptions of effectiveness, and certainty. However, there are limitations to the study that need to be acknowledged. First, the scope of the study was limited to only include allergy medicines, which is a low-risk medical issue. These effects may possibly differ if the materials were applied to a different medical issue, such as a medical issue that differs in its severity or novelty. Second, all responses provided by participants were self-reported, which can be open to recall bias. Third, the scales used in this study may be limited in how well they measure the constructs of interest; particularly, the effectiveness items used in this study were newly derived and created to match the specific stimulus materials employed. Although this was necessary due to the lack of suitable established measures, it may be that the scale was not robust in accounting for all potential variance in responses. Moreover, we found high overlap between the attitude and effectiveness belief items. This overlap may reflect attributes of the specific item selected or may be due to the sparse nature of the stimulus material. Taking these points together, future studies are required to follow up on these findings with different measures and stimulus materials. Finally, the sample was recruited through an online source, limiting the sample to only those with access to the Internet. Future research should aim to expand the scope of the study to other treatment domains and conditions to ensure the generalizability of the results described here.

## Conclusions

Two pre-registered experiments with multiple sets of stimuli identified strong and consistent effects of both framing and branding status on attitudes towards and effectiveness perceptions of allergy medications. Allergy medicines were evaluated more positively when presented using a positive rather than a negative frame, and branded medicines were rated more positively than generic medicines. Attitude certainty was also changed by the manipulations, although results on this variable differed somewhat across experiments. The results did not provide evidence that framing and branding status interact in influencing attitudes and effectiveness perceptions. Thus, these studies support the position that positive message-framing effects can be successfully employed to raise attitudes and perceptions of generic and branded medications.

## Supplementary Information

Below is the link to the electronic supplementary material.

Supplementary file1 (DOCX 14 KB)

## Data Availability

The deidentified data underlying the results presented in this study may be made available upon request from the corresponding author Emily Spotts.

## Code availability

Statistical analysis was performed on SPSS version 28.
