# Supplementary material for: The effect of attribute framing on beliefs and attitudes toward branded and generic medications
Source: J Behav Med. 2025 Mar 13;48(3):523–35. doi: 10.1007/s10865-025-00562-1 (PMC12078424; doi:10.1007/s10865-025-00562-1)
Supplement: Supplementary file 1 — Supplementary file1 (DOCX 14 KB) [file 10865_2025_562_MOESM1_ESM.docx]

Supplemental Materials

Medication Effectiveness Items

1. I would recommend [insert medication name here] to others.
2. I would use [insert medication name here] myself.
3. I am confident that [insert medication name here] work well.
4. I expect [insert medication name here] to work in the future.
5. I believe that [insert medication name here] are an effective medication for allergy symptoms.
6. Compared to other medications on the market, how effective do you think [insert medication name here] would be for treating allergy symptoms?
7. The experience of allergy symptoms would be completely resolved after using [insert medication name here].
